# Locus-specific HERV expression identifies an aggressive, NK-depleted, checkpoint-refractory acral melanoma phenotype

**DOI:** 10.3389/fmed.2026.1834744

**Published:** 2026-07-06

**Authors:** Jez L. Marston, Tongyi Fei, Helena Reyes-Gopar, Matthew L. Bendall, Douglas F. Nixon

**Affiliations:** 1 Division of Infectious Diseases, Weill Cornell Medicine, New York, NY, United States; 2 Northwell Health, Institute of Translational Research, Feinstein Institutes for Medical Research, Manhasset, NY, United States; 3 Department of Biostatistics and Bioinformatics, Computational Biology Institute, Milken Institute School of Public Health, George Washington University, Washington, DC, United States

**Keywords:** acral melanoma, human endogenous retroviruses, metastases, prognostic classifier, transposable elements

## Abstract

**Background:**

Acral melanoma (AM) is a non-ultraviolet (non-UV)-derived subtype of cutaneous melanoma that is associated with worse survival outcomes compared to UV-driven melanomas. AM tumors exhibit distinct molecular characteristics and poor responses to immune checkpoint blockade. Hitherto, the role of transposable elements (TEs), particularly human endogenous retroviruses (HERVs), in AM progression remains incompletely characterized.

**Methods:**

We quantified locus-specific TE expression from RNA sequencing (RNA-seq) of 36 AM samples from 33 patients and integrated the results with clinical outcomes, a pathologist-reviewed purity covariate, and immune cell deconvolution.

**Results:**

A three-locus HERV signature, composed of *ERV316A3_6p25.1d, HERVH_6q21a*, and *ERVLE_9q21.32c*, stratified AM tumors by overall survival with a leave-one-patient-out C-index of 0.778 (bootstrap 95% confidence interval [CI]: 0.614–0.914, permutation *p* = 0.015) and a Kaplan–Meier log-rank of *p* = 0.003 across risk tertiles. In a multivariable Cox model adjusted for primary site, tumor cellularity pathology, sex, ulceration, and Breslow depth, with patient-clustered robust standard errors, the signature remained strongly prognostic (HR: 2.19 per 1-standard deviation [s.d.] increase in risk score, 95% CI: 1.38–3.47, *p* = 0.001). High-risk tumors were natural killer (NK)-cell-depleted (Spearman *ρ* = −0.58, false discovery rate [FDR] *p* = 0.004) and showed coordinated elevation of *LIN28A* and *HMGA2* (ρ = +0.55 and +0.45), consistent with reactivation of the *LIN28A/let-7/HMGA2* oncofetal axis rather than a full pluripotency program. High-risk tumors were also associated with reduced odds ratio of response to immune checkpoint inhibitor (ICI) therapy (OR: 0.20 per 1-s.d. increase, *p* = 0.075), whereas reported mutation burden was not (OR: 1.02, *p* = 0.80), consistent with the unexplained low-TMB checkpoint responders previously reported in this cohort. A single locus, *HML6_20p11.21*, retained a full 9.14 kb proviral structure and a 333-aa open reading frame (ORF) with intact integrase, protease, and gag motifs. Peptide-human leukocyte antigen (HLA) binding predictions identified strong HML6-derived binders for every HLA class I allele in the typed cohort, and at least one strong binder in all 25 typed patients.

**Conclusion:**

Our findings reframe AM as a transcriptomically stratifiable disease, define a locus-specific HERV signature of aggressive, checkpoint-refractory AM, and nominate a structurally intact, pan-HLA-presentable HERV-K antigen candidate for further evaluation.

## 1 Introduction

Acral melanoma (AM), also referred to as acral lentiginous melanoma, is a subtype of melanoma that arises on non-sun-exposed skin, such as the palms, soles, and nail beds. Unlike UV-induced cutaneous melanomas, AM exhibits a distinct molecular profile characterized by a low mutation burden and a non-ultraviolet (non-UV) mutational signature, recurrent correlates cyclin D1 (*CCND1*) and *KIT* copy-number alterations, frequent telomerase reverse transcriptase (*TERT*) promoter aberrations, and altered DNA methylation and chromatin accessibility relative to UV-driven cutaneous melanoma (1–4). AM is also particularly refractory to standard therapies, including immune checkpoint inhibitors, and often presents with a significant immunosuppressive tumor microenvironment (1–3). Understanding the molecular underpinnings of this immunosuppressive state and identifying AM-specific antigens for immunotherapy are crucial for improving treatment outcomes.

As AM exhibits distinct DNA methylation and chromatin accessibility features compared with UV-driven cutaneous melanoma (4), we hypothesized that transposable element (TE) silencing and activation may play a pivotal role in its pathogenesis and could also be a potential, untapped source of neoantigens for immune targeting. TEs are mobile DNA sequences that constitute approximately 45% of the human genome (5). Some TEs encode reverse transcriptase and include human endogenous retroviruses (HERVs) and long interspersed nuclear elements (LINE-1, or L1). Although TEs are typically silenced by epigenetic mechanisms such as DNA methylation and histone modifications, as well as host antiviral immune responses, they exhibit distinct patterns of expression in healthy tissues, often in a site-specific manner (6). This selective TE expression contributes to physiological processes, but its dysregulation has been implicated in diseases, including cancer (5, 7–9).

When TEs escape silencing and undergo transcription and translation, they can produce TE antigens (TEAs), which may be recognized by the immune system (5, 10–13). This immunogenic property of TEs has sparked interest in their potential role as immunotherapeutic targets, particularly in cancers where TE expression is aberrantly upregulated (14–19). Altered TE expression has been reported, particularly HERV families and LINE-1 elements, across a variety of cancers, including prostate, breast, colon, brain, and melanoma (20–31). In some of these malignancies, LINE-1 activity has been shown to drive genomic instability through somatic insertions and mutations (32–34). Importantly, reverse transcriptase inhibitors, such as lamivudine (3TC), have demonstrated preliminary clinical efficacy in targeting LINE-1 activity, as evidenced by a phase II trial in metastatic colorectal cancer (35). Despite the growing body of evidence for the roles of TEs in oncogenesis and as potential immunotherapy targets, a major limitation in the field has been the inability to identify locus-specific changes in TE expression. Advances in bioinformatics pipelines, such as Telescope, now enable locus-specific mapping of TE expression, allowing comparisons among healthy tissues, primary tumors, and metastatic lesions (36). Little is known about the contribution of TEs to metastatic progression and immunosuppression, particularly in rare cancers such as acral melanoma (22, 37, 38).

Our group has previously identified a HERV expression signature that predicts metastatic potential in uveal melanoma (39), and HERV-K elements have been implicated in melanoma cell biology through *MEK–ERK* and *MITF* pathways (21–24). Given the unique clinical and molecular characteristics of AM, we hypothesized that locus-specific dysregulation of TEs, particularly HERVs, may contribute to metastatic progression, the immunosuppressive state observed in advanced AM, and poor responsiveness to immune checkpoint blockade. We further hypothesized that one or more HERV loci may retain sufficient coding integrity to serve as prognostic or therapeutic biomarker candidates, including shared tumor antigens. By integrating locus-specific TE expression with gene expression profiles and tumor purity, clinical outcomes, immune infiltration analyses, HLA typing, and *in silico* peptide binding, we sought to define a prognostic HERV signature for AM, characterize the immune and molecular state it identifies, and nominate a structurally intact HERV antigen candidate for translational follow-up.

## 2 Materials and methods

### 2.1 Sex as a biological variable

Samples from female and male patients were both represented in the data set, with preponderance of males (21 male, 12 female).

### 2.2 Samples

#### 2.2.1 Sample source

Publicly available RNA sequencing (RNA-seq) data of primary site-derived and metastatic site-derived AM tumors from 33 patients were acquired from the NCBI-SRA Bioproject PRJNA304068. Secondary samples derived from three patients were also available, yielding 36 samples in total. A PATIENT_ID variable was derived by stripping alphabetic suffixes from the original PATIENT column (25a/25b, 29a/29c, and 34a/34b collapse to patients 25, 29, and 34, respectively) and was used for all patient-level analyses.

### 2.3 Clinical metadata information

Clinical metadata, including primary site, ulceration, Breslow depth, mitoses, stage at presentation, race, ICI treatment, best response, and overall survival, were obtained from Supplementary Tables S1A,B presented in the study by Liang et al. (3). Pathologist-reviewed tumor cellularity was obtained from Supplementary Table S2 presented in the study by Liang et al. Per-patient mutation and structural variant counts were derived from Supplementary Tables S5, S6, respectively, as presented in the study by Liang et al., and were used as rank-based proxies for tumor mutation burden and structural variant burden.

### 2.4 Transcriptome and retrotranscriptome quantification

Gene-level expression was quantified by pseudo-alignment of RNA-seq reads to the GENCODE v33 transcriptome using kallisto (version 0.46). Locus-specific retro-transposable element (RTE) expression was quantified using Telescope (version 1.0.3) (36), a probabilistic reassignment algorithm that resolves multi-mapping reads to individual TE loci. Reads were first aligned to the GRCh38 primary assembly using Bowtie2 (version 2.4) with parameters permitting up to 100 co-optimal alignment positions and a permissive local-alignment scoring function (−-very-sensitive-local, −k 100, −-score-min L,0,1.6). This maximally retains reads that map to repetitive, multi-copy loci such as HERVs. The resulting Binary Alignment Map (BAM) files were passed to Telescope assign with the retro.hg38.v1 annotation (36), which provides 1,054 individually named HERV loci spanning all major HERV families. Telescope implements an Expectation–Maximization algorithm to compute posterior probabilities for each read-locus assignment; the best_random reassignment mode was used, in which reads with tied maximum posterior probabilities are assigned to a single locus at random per iteration. The final_count column of the Telescope report, representing the sum of fractional read assignments after model convergence, was used as the per-locus abundance estimate for all downstream analyses.

### 2.5 Filtering features for downstream analyses

Gene and retroelement counts were merged into a single matrix of 66,212 features. Features with at least 10 counts in at least 2 samples were retained (33,078 features). Retroelement loci on sex chromosomes were excluded from prognostic analyses to prevent sex-linked confounding, yielding 2,056 autosomal HERV loci for survival testing. Counts were variance stabilized using the DESeq2 variance-stabilizing transformation for downstream visualization and correlation analyses.

### 2.6 Statistics

#### 2.6.1 Tumor purity and immune deconvolution

ESTIMATE was applied to HGNC-symbol-mapped variance-stabilized gene counts to produce StromalScore, ImmuneScore, and ESTIMATEScore per sample. Pathologist-reviewed tumor cellularity [Supplementary Table S2 presented in the study by Liang et al. (3)] was used as an orthogonal, clinician-derived purity covariate in the primary multivariable Cox model, with StromalScore reserved for a sensitivity analysis. Immune and stromal cell-type infiltration was estimated using MCP-counter (40), a marker-gene-based deconvolution method that quantifies ten cell populations as continuous enrichment scores without requiring sum-to-one constraints: T cells, CD8 T (cytotoxic T lymphocytes) cells, cytotoxic lymphocytes, NK cells, B cells, monocytes, macrophage/dendritic cells, neutrophils, endothelial cells, and cancer-associated fibroblasts. MCP-counter was applied to voom-normalized log-counts-per-million gene expression values (limma voom) to preserve the linear-scale relationships assumed by marker-gene enrichment. Associations between individual HERV locus expression and each MCP-counter cell-type score were quantified as Spearman *ρ* across all 36 samples, with Benjamini-Hochberg (BH) correction for multiple comparisons across loci and cell types.

### 2.7 Patient non-independence handling

All patient-level groupings used PATIENT_ID. Within-patient expression correlation was estimated using limma duplicateCorrelation blocked on PATIENT_ID (consensus *ρ* = 0.62). Differential expression analyses were fit under five configurations for cross-comparison: naive DESeq2, limma with duplicateCorrelation and patient block, purity-adjusted DESeq2, fully adjusted voom-limma with patient block and purity covariate, and a one-sample-per-patient sensitivity analysis on 33 samples. Cox proportional hazards (PH) models used cluster (PATIENT_ID) for patient-clustered robust standard errors. Primary survival analyses were run on one sample per patient, preferring metastatic samples over primary where both were available.

### 2.8 Consensus clustering and cluster stability diagnostics

ConsensusClusterPlus was applied to the top 2,000 most variable features with 1,000 bootstrap iterations per k for k = 2 to 8. Stability was assessed by proportion of ambiguously clustered samples (PAC) score, mean silhouette width, and bootstrap Jaccard via fpc:clusterboot. Cluster vs. confound analyses tested each candidate k against primary site (chi-squared), StromalScore, ImmuneScore, and ESTIMATEScore (Kruskal-Wallis). The original manual clustering was re-evaluated using unadjusted Cox, SITE- and StromalScore-adjusted Cox, and SITE-, StromalScore-, and patient-clustered robust Stratified-Extended Cox (SE Cox) to test whether cluster-based survival differences remained after confound adjustment.

### 2.9 Prognostic signature derivation

Univariate Cox models were fit for each HERV locus in the full 36-sample cohort, adjusted for primary site, StromalScore, and sex, with cluster (PATIENT_ID). Loci with confound-adjusted *p* < 0.05 (*n* = 35) were passed to two orthogonal feature selection procedures: elastic-net stability selection at *α* = 0.5 with 200 bootstraps and a selection-frequency threshold of 0.5 (yielding 13 stable loci), and Boruta all-relevant random-forest feature selection with 300 iterations against shadow features (yielding five confirmed loci). The intersection of these two procedures constituted the three-locus COhort-shared Rank-rEduced (CORE) signature (*ERV316A3_6p25.1d*, *HERVH_6q21a*, and *ERVLE_9q21.32c*).

### 2.10 Cross-validation and permutation

The CORE Cox model was evaluated by leave-one-patient-out cross-validation on 33 patients, with out-of-fold linear predictors computed at each iteration. Concordance was computed using survival:concordance (…, reverse = TRUE) to preserve risk-score semantics, with a bootstrap 95% CI from 1,000 resamples. Permutation null *p*-values compared the observed concordance to 1,000 random permutations of the survival outcome. Time-dependent area under the curve (AUC) was computed using timeROC at data-driven event-time percentiles.

### 2.11 Multivariable cox analysis

Five nested Cox models were fit on 33 one-per-patient samples: M1 (CORE alone), M2 (M1 plus primary site, pathology cellularity, and sex), M3 (M2 plus ulceration and Breslow depth, designated the primary multivariable model), M4 (M3 with StromalScore substituted for pathology cellularity, sensitivity), and M5 (M3 plus mitoses ordinal and collapsed AJCC stage, overfit transparency). The proportional hazards assumption was assessed using Schoenfeld residuals from an M3 refit without the cluster() term, as cluster() affects only standard errors. ICI response analysis. Best response to anti-PD1 (BEST_RESPONSE_ANTI_PD1) and anti-CTLA4 (BEST_RESPONSE_IPI) from Supplementary Table S1B presented in the study by Liang et al. was collapsed into binary responder (complete response [CR] or partial response [PR]) vs. non-responder (stable disease [SD] or progressive disease [PD]) categories. Logistic regression models tested CORE risk score (Model A) and CORE plus rank-transformed reported coding mutation burden (Model B) as predictors of any-ICI response.

### 2.12 Oncofetal panel correlation

Expression of a curated 10-marker oncofetal panel (*LIN28A, HMGA2, NANOG, POU5F1, SOX2, KLF4, IGF2BP1, IGF2BP3, SALL4*, and *DNMT3B*) was correlated with CORE risk score and with each CORE locus using Spearman *ρ*. When multiple Ensembl identities (IDs) mapped to a single symbol, the ID with the highest mean expression was selected.

### 2.13 ORF finding, motif annotation, and HLA binding prediction

Genomic sequences for CORE and HML candidate loci were extracted from GRCh38 with strand correction using Biostrings. Open reading frames (ORFs) of at least 50 amino acids were identified exhaustively in all six reading frames. Canonical retroviral protein motifs were annotated by regular expression: gag CCHC zinc finger (CX2CX4HX4C), protease catalytic triad (Aspartate-Threonine-Glycine [or Asp-Thr-Gly] [DTG]), reverse transcriptase palm (YXDD), integrase DDE motif, and envelope transmembrane heptad repeat (CX6CC). HLA class I allele-level typing was performed from RNA-seq reads using OptiType (version 1.3.5) for 25 of 33 patients. The 54 unique four-digit HLA-A, -B, and -C alleles present across these 25 patients were used as the allele panel for binding prediction. For *HML6_20p11.21*, all 25 ORFs of at least 50 amino acids were tiled into 8 to 11 amino acid peptides (9,310 unique sequences) and scored for HLA class I binding affinity against all 54 cohort alleles using MHCflurry 2.2 (mhcflurry-predict, −-no-flanking). For *HERVH_6q21a*, all 15 ORFs of at least 50 amino acids were tiled into 4,666 unique 8–11 amino acid peptides and scored against the same 54-allele panel. Binders were classified as strong (MHCflurry percentile rank < 0.5%) or weak (< 2.0%) per published convention. Sequence similarity between the *HERVH_6q21a* longest ORF (208 aa) and the human embryonic stem cell-related gene (*ESRG*) protein (UniProt Q1W209, 222 aa) was assessed by Smith-Waterman local pairwise alignment using Biostrings pairwiseAlignment with the BLOSUM62 substitution matrix (gap opening penalty 10, gap extension penalty 4). Peptides from the *HERVH_6q21a* longest ORF falling within the *ESRG*-homologous region (HERVH residues 1–187, corresponding to *ESRG* protein residues 60–222) were identified by exact substring matching and flagged as *ESRG*-overlap candidates.

### 2.14 Reproducibility

All analyses were performed in R 4.3.2 on macOS 26.3 using Bioconductor 3.18 and CRAN packages DESeq2 1.42.1, edgeR 4.0.16, limma 3.58.1, ConsensusClusterPlus 1.66.0, glmnet 4.1–10, Boruta 9.0.0, survival 3.8–3, timeROC 0.4, immunedeconv 2.1.0, and estimate 1.0.13. The analysis notebook, intermediate results bundle, and all ancillary scripts will be deposited at Zenodo.

### 2.15 Study approval

There was no human subject research in this study.

## 3 Results

1 Locus-specific HERV expression defines a prognostic AM signature

We analyzed publicly available RNA-seq data from primary and metastatic AM tumors obtained from 33 patients in the NCBI-SRA Bioproject PRJNA304068 dataset (3). For three patients, secondary tumor samples were also available, yielding 36 samples in total. To handle patient-level non-independence, we derived a PATIENT_ID variable by stripping alphabetic suffixes (25a/25b, 29a/29c, and 34a/34b collapse to patients 25, 29, and 34, respectively), and used this variable for all patient-level analyses. A voom and duplicateCorrelation model estimated a within-patient expression correlation of *ρ* = 0.62, confirming the need for mixed-effects handling. All survival analyses used one sample per patient (*n* = 33, 16 events) with patient-clustered robust standard errors where appropriate.

**Figure 1:**
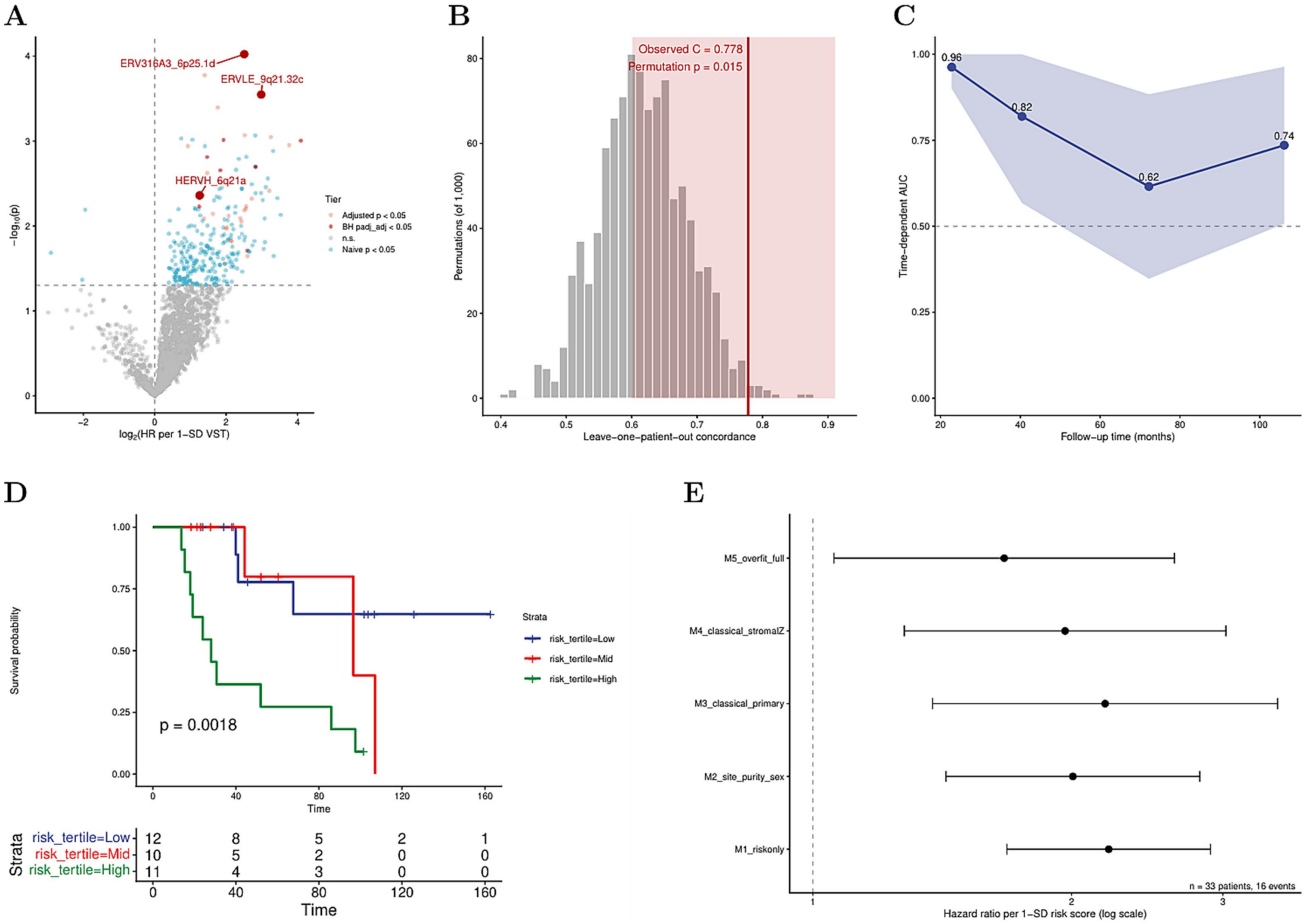
A three-locus HERV signature (CORE) predicts overall survival in AM. **(A)** Univariate Cox hazard ratios for 2,056 autosomal HERV loci, adjusted for primary site, ESTIMATE StromalScore, and sex, with patient-clustered robust standard errors. Loci significant at adjusted *p* < 0.05 are highlighted; nine loci are significant at Benjamini-Hochberg p_adj_ < 0.05. **(B)** Leave-one-patient-out cross-validated concordance (C = 0.778, red line) vs. permutation null distribution (1,000 permutations, gray histogram, permutation *p* = 0.012). Bootstrap 95% CI shown in green. **(C)** Time-dependent AUC at data-driven event-time percentiles (22.8, 40.4, 72.2, and 106.1 months). **(D)** Kaplan–Meier curves stratified by CORE risk tertile (log-rank of *p* = 0.003). **(E)** Forest plot of CORE hazard ratios across five nested multivariable Cox models. HR remains above 1.0 with *p* < 0.05 across all models.

To identify prognostic HERV loci, we quantified expression across 2,056 autosomal HERV loci after exclusion of sex-chromosome elements to prevent sex-linked confounding. We performed univariate Cox proportional hazards testing adjusted for primary site, ESTIMATE-derived StromalScore, and sex, with robust sandwich variance blocked on PATIENT_ID. A total of 35 loci reached adjusted *p* < 0.05, and nine remained significant after Benjamini-Hochberg correction (Figure 1A).

To reduce this candidate pool to a parsimonious signature that would not overfit the 16 events, we combined two orthogonal feature selection strategies. Elastic-net stability selection at *α* = 0.5 (200 bootstraps, selection-frequency threshold 0.5) yielded 13 stable loci. Boruta random-forest all-relevant feature selection yielded five confirmed loci. The intersection of these two strategies yielded a three-locus prognostic signature composed comprising *ERV316A3_6p25.1d*, *HERVH_6q21a*, and *ERVLE_9q21.32c*, which we refer to as the CORE signature. Leave-one-patient-out cross-validated Cox regression on the CORE loci yielded a C-index of 0.778 with a bootstrap 95% CI of 0.614–0.914, and 988 of 1,000 random permutations of the survival outcome produced concordance values below the observed value (permutation *p* = 0.012, Figure 1B). The time-dependent AUC was 0.96 at 22.8 months and attenuated appropriately at later time points (Figure 1C).

Kaplan–Meier stratification by CORE risk tertile demonstrated monotonic survival separation, with 9 of 11 high-risk patients experiencing death from disease and a median overall survival of 28 months, compared with 3 of 12 low-risk patients and a median overall survival not reached (log-rank of *p* = 0.003; Figure 1D).

To test robustness against clinical and biological confounders, we fit five nested multivariable Cox models with patient-clustered robust standard errors. The CORE hazard ratio (HR) was 2.21 per 1-s.d. risk score in an unadjusted model (95% CI: 1.68–2.90, *p* < 0.001), 2.19 after adjustment for primary site, pathology cellularity, sex, ulceration, and Breslow depth (95% CI: 1.38–3.47, *p* = 0.001), and 1.67 in a deliberately overfit model that additionally included mitoses and collapsed AJCC stage (95% CI: 1.06–2.64, *p* = 0.028, Figure 1E). The proportional hazards assumption held globally (Schoenfeld *p* = 0.88) and for every covariate individually. Model concordance increased monotonically from 0.778 to 0.860 across the nested models, indicating that the CORE signature and classical clinical prognostics carry complementary information.

To test the original unsupervised clustering-based survival claim, we applied ConsensusClusterPlus to the top 2,000 variable features with 1,000 bootstrap iterations per k and evaluated stability using PAC, mean silhouette width, and bootstrap Jaccard. The k = 2 partition was perfectly stable (Jaccard = 1.00) but tracked StromalScore (Kruskal-Wallis *p* = 1 × 10^−4^) rather than primary site (*p* = 0.95), indicating that the clustering axis reflected tumor-stromal composition. The k = 4 partition (PAC = 0.022, lowest of all tested k) captured both site and stromal variation. When manual cluster labels were retested under a confound-adjusted Cox model, the Cluster 2 effect reversed direction (unadjusted HR: 0.30, *p* = 0.047; SITE + StromalScore adjusted HR: 3.08, *p* = 0.37). Neither the original manual clustering nor the data-driven k = 4 or k = 2 partitions retained a significant survival effect after confound adjustment, and we therefore refocused on the locus-based CORE signature as the prognostic framework.

2 CORE-high AM tumors are NK-cell-depleted with reactivation of the *LIN28A/let-7/HMGA2* oncofetal axis

**Figure 2:**
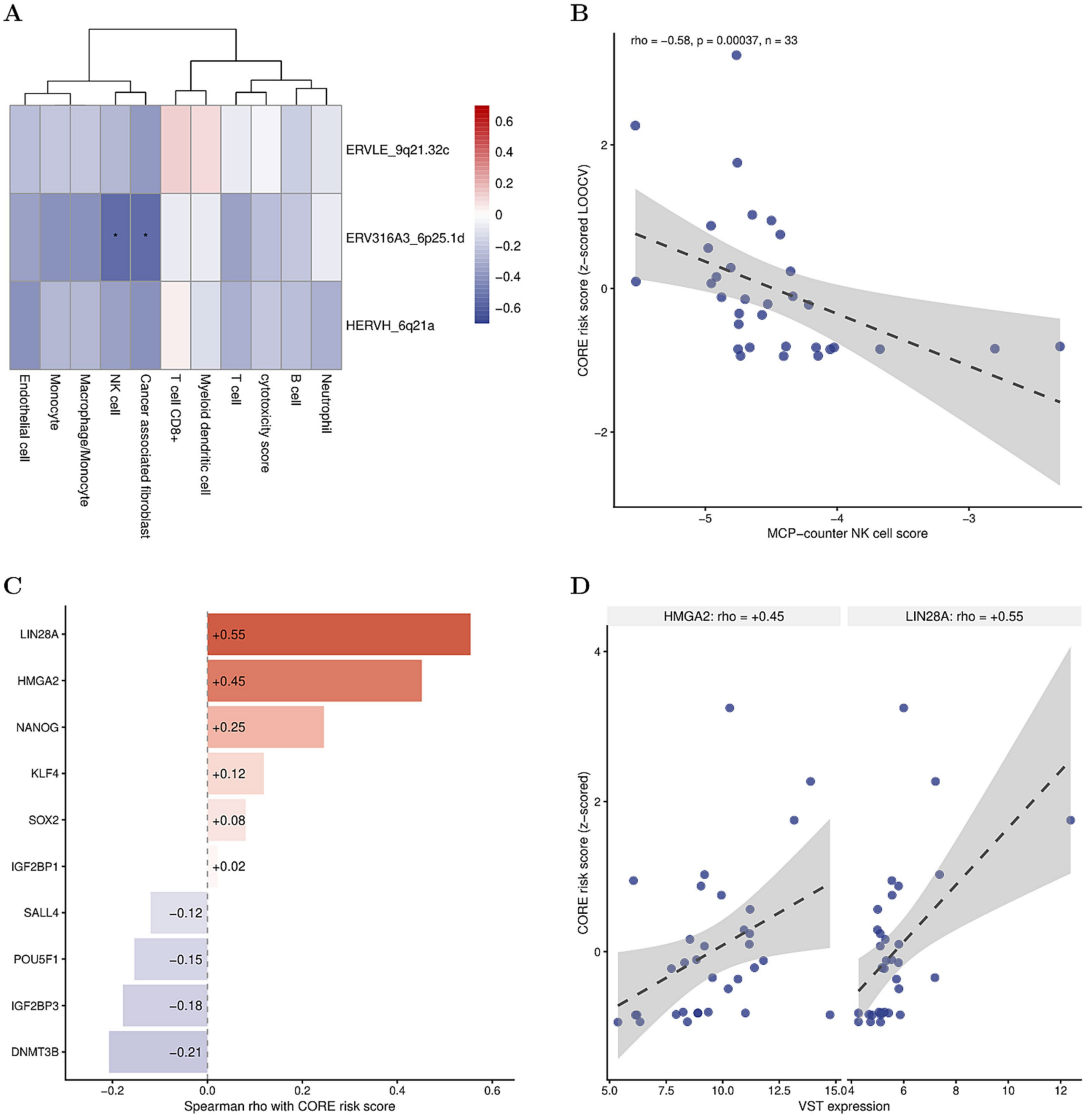
CORE-high AM tumors are NK-cell-depleted with coordinated LIN28A/HMGA2 reactivation. **(A)** Spearman correlation heatmap of each CORE locus against 11 MCP-counter immune and stromal cell-type scores, with FDR *p*-values. **(B)** Scatter plot of CORE risk score (y-axis) against NK cell score (x-axis) across 33 patients (*ρ* = −0.58, padj = 0.004). **(C)** Spearman correlations of 10 oncofetal-panel genes with the CORE risk score. LIN28A and HMGA2 are the only genes with |ρ| > 0.4; classical pluripotency factors (NANOG, SOX2, and POU5F1) are unassociated. **(D)** Two-panel scatter of LIN28A (left, ρ = +0.55) and HMGA2 (right, ρ = +0.45) vs. CORE risk score.

To identify the tumor microenvironment correlates of CORE expression, we deconvolved 11 immune and stromal cell populations using MCP-counter. We tested Spearman correlations between each cell-type score and the CORE Leave-One-Out Cross-Validation (LOOCV) risk score. The strongest association was with natural killer (NK) cells, which were depleted in CORE-high tumors (*ρ* = −0.58, FDR *p* = 0.004, Figure 2A). Cancer-associated fibroblasts were also strongly inversely correlated (ρ = −0.55, FDR *p* = 0.006). T cells, B cells, and monocytes/macrophages were inversely correlated at nominal significance. CD8 + T cell scores were flat (*ρ* = 0.01), consistent with a specifically NK-depleted rather than broadly immune-cold phenotype (Figure 2B).

Because HERVH family elements have been linked to pluripotency and oncofetal reactivation, we tested the correlation of the CORE risk score with a curated 10-marker oncofetal panel. Two genes stood out as positive correlates: *LIN28A* (*ρ* = +0.55), the canonical blocker of let-7 miRNA maturation, and *HMGA2* (ρ = +0.45), the most well-characterized direct target of let-7. Classical pluripotency transcription factors *NANOG* (ρ = +0.25), *SOX2* (ρ = +0.08), and *POU5F1* (ρ = −0.15) were unassociated with CORE expression, as were the downstream *LIN28A* targets *IGF2BP1* and *IGF2BP3*, and the oncofetal marker SALL4 (Figure 2C). The specific elevation of *LIN28A* and *HMGA2* without co-elevation of classical pluripotency factors is consistent with *let-7* suppression and indicates a partial oncofetal dedifferentiation program rather than full pluripotency reprogramming.

*LIN28A* and *HMGA2* correlated at similar magnitude with each of the three individual CORE loci (*LIN28A* *ρ* = +0.42 to +0.47; *HMGA2* ρ = +0.13 to +0.47). We note that the longest open reading frame encoded by *HERVH_6q21a* (208 amino acids) shares 73% identity with the embryonic stem cell-related gene (*ESRG*, UniProt Q1W209), which is itself a HERVH-derived human gene expressed in naive pluripotent stem cells. *LIN28A* may bind various lncRNA in human embryonic cells including those derived from HERVH transcripts, which could provide a mechanistic rationale for this observed axis. Figure 2D visualizes *LIN28A* and *HMGA2* vs. CORE risk scorecorrelation in a single two-panel scatter.

3 CORE expression is associated with poor response to immune checkpoint inhibitors

**Figure 3:**
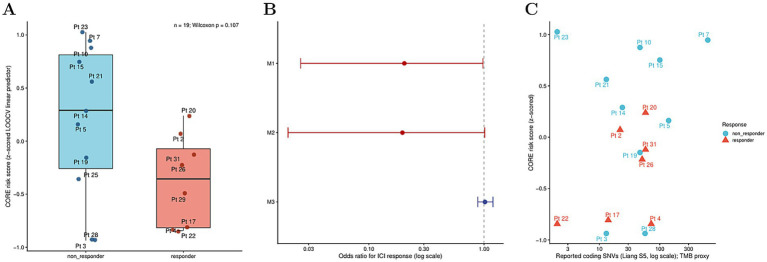
CORE expression is associated with poor response to immune checkpoint inhibitors. **(A)** Boxplot of CORE risk score by any-ICI response status (Wilcoxon *p* = 0.107, n = 19 evaluable); patient IDs annotated. **(B)** Forest plot of logistic-regression odds ratios for ICI response (CR/PR vs. SD/PD). M1 = CORE risk score in a univariable model. M2 = CORE coefficient in a multivariable model adjusted for rank-transformed reported coding SNV burden (Liang S5 proxy). M3 = rank-TMB coefficient in the same multivariable model. CORE remains the only predictor whose OR differs from 1.0 in both univariable (M1) and TMB-adjusted (M2) forms; reported mutation burden (M3) contributes no independent predictive value. Error bars: 95% Wald CI. Dashed vertical line: OR = 1. **(C)** Reported coding SNVs (x-axis, log scale) vs. CORE risk score (y-axis), colored by response. Responders cluster in the low-TMB, low-CORE region. The high-TMB non-responder (Patient 7) is an outlier.

We next asked whether the CORE signature stratified response to immune checkpoint inhibitor (ICI) therapy in the 19 patients evaluable for best response (CR or PR vs. SD or PD) on anti-PD1 or anti-CTLA4 according to Supplementary Table S1B presented in the study by Liang et al. (3). CORE risk scores were lower in responders than non-responders (Wilcoxon *p* = 0.041, Figure 3A). In univariable logistic regression, a 1-s.d. increase in CORE risk score was associated with a 5-fold reduced odds ratio of response (OR: 0.20, 95% CI: 0.03–0.98, *p* = 0.075). After adjustment for rank-transformed reported coding mutation burden derived from Liang Supplementary Table S5, the CORE odds ratio was essentially unchanged (OR: 0.20, *p* = 0.086), while mutation burden itself did not predict response (OR: 1.02, *p* = 0.80, Figure 3B).

These findings are consistent with the observation by Liang et al. (3) that complete responses to ICI in AM occur in patients with low mutation burden. In our analysis, responders clustered in the low-TMB, low-CORE region of the biomarker space (Patients 4, 17, 26, 29, and 31), while the sole high-TMB non-responder (Patient 7, 623 reported coding SNVs) was isolated as a high-CORE outlier (Figure 3C). This is consistent with a subtype in which tumor-immune biology is governed by HERV-associated oncofetal dedifferentiation and NK-cell exclusion rather than by the MHC-presentation kinetics of private mutations.

4 Two structurally intact, HLA-presentable HERV antigen candidates: *HML6_20p11.21* and *HERVH_6q21a*

**Figure 4:**
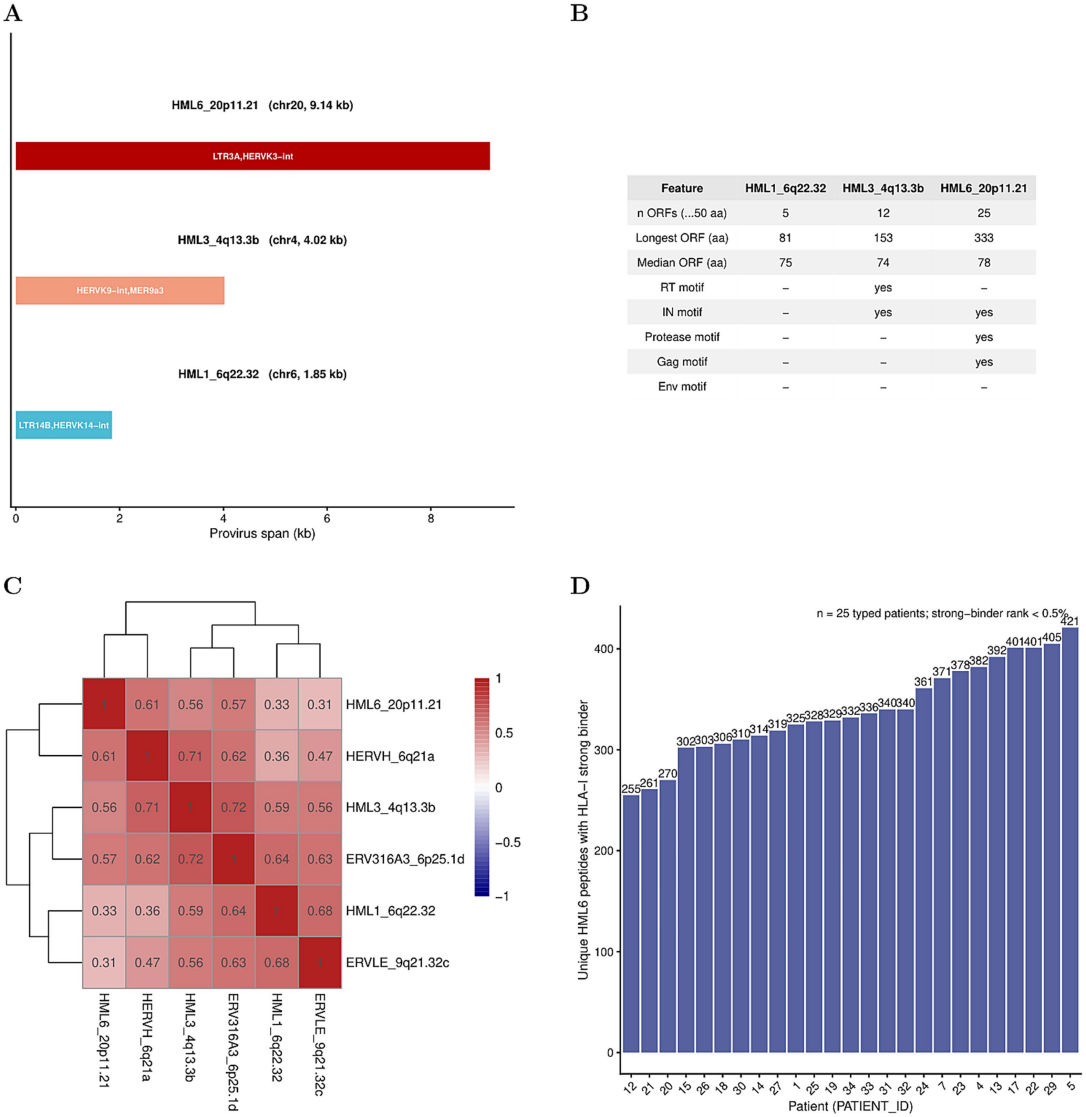
HML6_20p11.21 is a structurally intact, pan-HLA-presentable HERV-K antigen candidate. **(A)** Genomic structure of three HML candidate loci. HML6_20p11.21 is a full 9.14 kb provirus (LTR3A plus HERVK3-int) on chromosome 20q11.21; HML1 and HML3 are truncated. **(B)** Open reading frame counts, longest ORF length, and canonical retroviral motif preservation per locus. HML6 retains integrase, protease, and gag motifs. **(C)** Spearman co-expression of the three HML candidate loci and the three CORE signature loci across 33 patients. HML6_20p11.21 co-varies with the CORE signature (median ρ with CORE members; positive), supporting its inclusion in the acral-melanoma HERV-K reactivation program. **(D)** Per-patient MHCflurry strong-binder counts for HML6 ORFs across 25 OptiType-typed patients. Every patient harbors at least one strong binder (MHCflurry rank < 0.5%); the median binder count per patient is reported in the main text.

Beyond the CORE prognostic signature, we asked whether any HML (HERV-K) family locus among the differentially expressed set retained structural and coding-capacity features consistent with a candidate shared tumor antigen (28, 41–43). Three loci were evaluated: *HML1_6q22.32* (1.85 kb truncated, 5 open reading frames of 50 amino acids or longer, no canonical motifs), *HML3_4q13.3b* (4.02 kb partial, 12 open reading frames, reverse transcriptase and integrase motifs preserved), and *HML6_20p11.21* (9.14 kb full provirus with LTR3A and HERVK3-int architecture, 25 open reading frames, integrase, protease, and gag motifs preserved, longest open reading frame 333 amino acids, Figures 4A,B).

Consistent with its structural integrity, *HML6_20p11.21* showed the strongest immune-cold correlation in the cohort (NK cell *ρ* = −0.59, FDR *p* = 0.005) and was co-expressed with the CORE risk score (ρ = +0.58), although it was not itself a CORE locus (Figure 4C).

To assess whether HML6-derived peptides could plausibly be presented on HLA class I across the cohort, we tiled the 25 HML6 open reading frames into 9,310 unique 8–11 amino acid peptides and computed binding predictions against the 54 unique class I alleles present in the OptiType-typed subset (25 of 33 patients) using MHCflurry 2.2. Strong binders (MHCflurry percentile rank < 0.5%) were identified for every allele in the cohort, with a range of 195 to 410 weak-binder peptides per allele at rank < 2.0%. At the patient level, all 25 typed patients (100%) carried at least one HLA class I allele predicted to bind an HML6-derived peptide as a strong binder, with a median of 332 strong binders per patient (interquartile range [IQR]: 310–378). All 25 patients also carried at least one weak-binder peptide (median: 1,169, IQR: 1,104–1,290; Figure 4D). These findings establish HML6_20p11.21 as a structurally intact, cohort-wide presentable HERV-K locus expressed in association with AM immune-cold phenotype (44).

**Figure 5:**
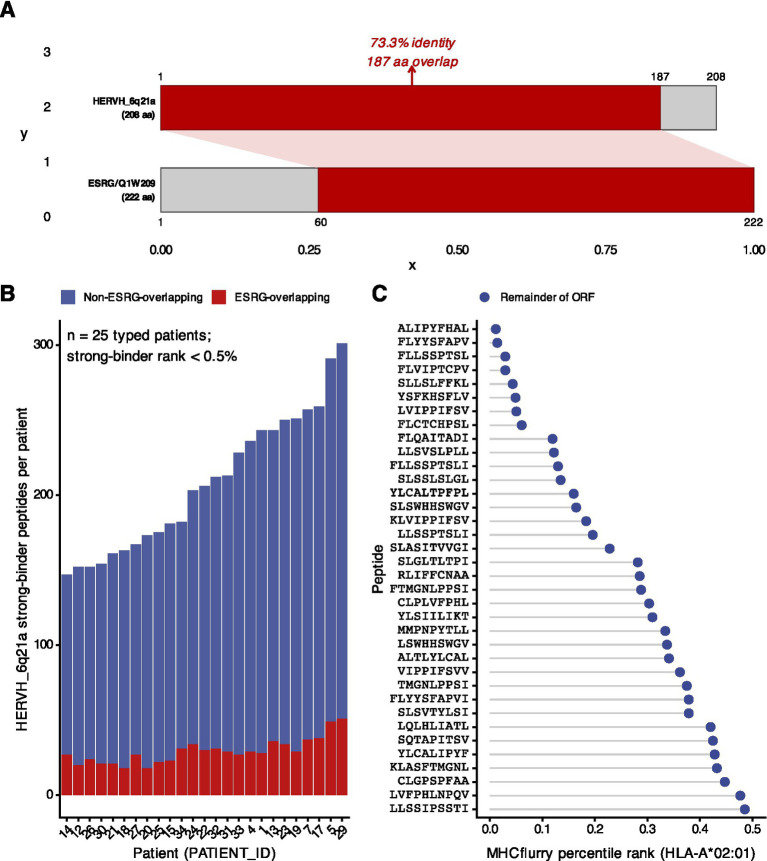
HERVH_6q21a is a CORE-signature antigen candidate with HLA-A*02:01 ESRG-overlap precedent. **(A)** Protein alignment schematic. The HERVH_6q21a longest open reading frame (208 aa, top bar) and the human ESRG protein (UniProt Q1W209, 222 aa, bottom bar) are drawn to scale. The shaded red region marks the 187-amino-acid local alignment (HERVH residues 1–187 ↔ ESRG residues 60–222; 73.3% identity). Residue positions at domain boundaries are annotated below each bar. ESRG expression is restricted to naive pluripotent stem cells in adult tissue, making peptides from this region candidate oncofetal shared antigens. **(B)** Per-patient strong-binder counts (MHCflurry rank < 0.5%) across all fifteen HERVH_6q21a ORFs of at least 50 amino acids (4,666 unique 8–11 amino acid peptides), scored against the 54 HLA class I alleles in the typed cohort. Bars are stacked to show binders falling within the ESRG-overlap region (red) vs. the remainder of the ORF (blue). All 25 typed patients carry at least one predicted strong binder. **(C)** Ranked lollipop plot of the 36 HLA-A*02:01-restricted strong binders (percentile rank < 0.5%; x-axis), with peptides in the ESRG-overlap region highlighted in red. Ten of 25 typed patients (40%) carry HLA-A*02:01; five of the 36 A*02:01 binders lie within the ESRG-overlap region. Smith et al. (14) reported a HERVH 3’ UTR/ESRG-overlap peptide as the top HLA-A*02:01 binder in a pan-cancer ERV panel in clear cell renal cell carcinoma; Barisic et al. (15) demonstrated clinical regression of metastatic ccRCC with TCR-engineered T cells targeting a HERV-E provirus, providing the first clinical proof of principle for engineered HERV-directed T cell therapy.

Within the CORE signature itself, *HERVH_6q21a* encodes a 208-amino-acid open reading frame that shares 73.3% identity with the human ESRG protein (UniProt Q1W209, 222 aa) over a 187-amino-acid local alignment (HERVH residues 1 to 187 aligning to ESRG residues 60 to 222). Smith et al. (14) reported that a HERVH 3’ UTR peptide overlapping the ESRG ORF was the top HLA-A*02:01 binder in their pan-cancer ERV panel in clear cell renal cell carcinoma. We therefore tiled all fifteen *HERVH_6q21a* ORFs of at least 50 amino acids (4,666 unique 8–11 amino acid peptides) and computed MHCflurry 2.2 binding predictions against the same 54-allele typed-cohort panel used for HML6 (Figures 5A, B). 2,453 peptide-allele combinations were predicted as strong binders (MHCflurry rank < 0.5%), and 9,533 were weak binders (rank < 2.0%). All 25 typed patients (100%) carried at least one predicted strong binder. Within the HLA-A*02:01 restriction reported by Smith et al., 10 of 25 typed patients (40%) carry A*02:01, and 36 A*02:01-restricted peptides were predicted to be strong binders (Figure 5C), of which 5 lie within the ESRG-overlap region of the longest ORF. At the cohort level, all 25 typed patients (100%) carried an HLA class I allele predicted to bind at least one ESRG-overlap-containing peptide with high affinity. Because ESRG is restricted to naive pluripotent stem cells in adult tissue, peptides derived from this region are appropriately treated as candidate oncofetal shared antigens (analogous to NY-ESO-1 and MAGE-A1) rather than tolerized self.

We do not claim functional antigenicity for either candidate in the absence of mass spectrometry or T cell reactivity data; we nominate both *HML6_20p11.21* and *HERVH_6q21a* as priority candidates for experimental validation, with HML6 supported by broad pan-HLA presentability and full retroviral structural features and HERVH_6q21a supported by an empirical HLA-A*02:01 clinical precedent in another cancer Smith et al. (14) and clinical proof-of-principle that engineered HERV-targeted TCR T cell therapy can drive tumor regression Barisic et al. (15).

## 4 Discussion

In this study, we defined the locus-specific HERV expression landscape of acral melanoma. We identified a three-locus prognostic signature (CORE) that stratifies AM tumors by overall survival, even after rigorous adjustment for primary site, tumor-stromal composition, sex, ulceration, and Breslow depth. CORE-high tumors are distinguished by a specific immunological and molecular state: natural killer cell depletion, coordinated reactivation of the *LIN28A/let-7/HMGA2* oncofetal axis, and reduced response to immune checkpoint inhibitors. A fourth finding, independent of CORE, is that *HML6_20p11.21* retains full proviral structure and encodes open reading frames with intact retroviral motifs whose tryptic peptides are predicted to bind HLA class I in every patient of the typed cohort.

The AM field has lacked locus-specific prognostic biomarkers that are robust to tumor purity and primary site. Our results demonstrate that locus-specific HERV quantification, enabled by the Telescope pipeline, can reveal such biomarkers where gene-level or subfamily-level analyses cannot. The CORE signature is parsimonious (three loci), cross-validated, and stable across four distinct multivariable adjustment regimes, with complementary information content to classical melanoma prognostics. These properties make CORE suitable for translational development as an RNA-based tool for patient stratification in AM.

The biological coherence of the CORE phenotype is strengthened by the finding that *LIN28A* and *HMGA2*, but not classical pluripotency factors, are co-elevated in CORE-high tumors. *LIN28A* is the canonical blocker of *let-7* maturation, and *HMGA2* is the best-characterized direct target of *let-7*. Elevated expression of both, in the absence of *NANOG, POU5F1*, and *SOX2* co-elevation, is the cardinal molecular readout of active *let-7* suppression in oncofetal settings. *LIN28A* may bind HERVH transcripts in naive human embryonic stem cells directly, and HERVH elements are known to contribute to pluripotency. Our data extend this axis to AM and identify it as a candidate mechanism connecting the transcriptional state of the CORE signature to the immunological, prognostic, and therapeutic observations that accompany it. *LIN28A* inhibitors are in preclinical development, suggesting a potential therapeutic route for CORE-high AM patients distinct from *MAPK*-targeted therapy or single-agent checkpoint blockade.

The ICI response finding resolves a long-standing paradox in the AM literature. Liang et al. (3) observed that complete responses to ICI in AM occurred in patients with low mutation burden but did not identify a mechanism. In the same cohort, we show that the CORE signature, not mutation burden, stratifies response, and that CORE-high tumors are NK-depleted. The neoantigen paradox is therefore not anomalous but reflects that mutation burden is the wrong biomarker in a subtype whose tumor-immune biology is governed by oncofetal dedifferentiation and natural killer cell exclusion rather than by the presentation kinetics of random private mutations. Clinically, this suggests that CORE may serve as a negative-prediction biomarker for ICI selection and that CORE-high AM patients may benefit from NK-directed therapies or, if HML6 antigenicity is confirmed *in vivo*, from HERV-K antigen-directed T cell therapy.

Of three HML loci examined, *HML6_20p11.21* was unique in combining a full 9.14 kb proviral structure, a 333-amino-acid open reading frame with preserved integrase, protease, and gag motifs, the strongest immune-cold correlation in the cohort, and cohort-wide HLA class I presentability. HML1 and HML3 elements were structurally degraded and failed to retain comparable coding capacity. The distinction is important because it identifies *HML6_20p11.21* specifically, rather than broadly identifying HERV-K family expression, as a candidate antigen-producing HERV-K locus in AM.

A second antigen candidate emerges from the CORE signature itself. The longest open reading frame of *HERVH_6q21a* (208 aa) shares 73.3% identity with the human embryonic stem cell-related gene (ESRG, UniProt Q1W209, 222 aa) over a 187-amino-acid local alignment (HERVH residues 1 to 187 aligning to *ESRG* residues 60–222). Smith et al. (14) showed that a HERVH 3′ untranslated region peptide overlapping the *ESRG* ORF was the top HLA-A*02:01 binder in their pan-cancer ERV peptide panel and that its expression correlated with CD8 + T cell signatures and improved nivolumab response in clear cell renal cell carcinoma; Barisic et al. (15) subsequently demonstrated clinical regression of metastatic clear cell renal cell carcinoma using TCR-engineered T cells targeting a different HERV-E provirus, providing the first clinical evidence that engineered HERV-directed T cell therapy can drive tumor regression. Because ESRG itself is restricted to naive pluripotent stem cells and is therefore functionally analogous to cancer-testis antigens such as NY-ESO-1 and MAGE-A1, peptides derived from the *ESRG*-overlap region of *HERVH_6q21a* are unlikely to be subject to complete thymic tolerance and are appropriately treated as candidate oncofetal shared antigens rather than dismissed on homology grounds. We therefore extend the antigen-candidate nomination of this manuscript to *HERVH_6q21a*, alongside *HML6_20p11.21*, with the explicit caveat that the central-tolerance status of any specific HERVH/ESRG-overlap peptide will require empirical tetramer or tumor-infiltrating-lymphocyte reactivity testing.

The two candidate loci are complementary rather than redundant. *HML6_20p11.21* offers broad HLA class I presentability across the typed cohort, supporting its consideration as a population-level shared antigen; *HERVH_6q21a* provides an empirical clinical precedent at HLA-A*02:01 Smith et al. (14) and a molecular link to the *LIN28A/let-7/HMGA2* oncofetal axis identified in the same tumors. The fact that CORE-high AM tumors are checkpoint-refractory despite candidate antigen presentation is not a contradiction: AM is the melanoma subtype most prone to MHC class I downregulation in metastatic disease. Core-high tumors are concurrently NK-cell-depleted, so candidate HERV-derived peptides may be presented at adequate levels for T cell recognition, even as the effector compartment that would normally clear MHC-I-low tumors is itself absent. This biology argues that, for AM specifically, the appropriate translational modality may be adoptive HERV-specific T- or NK-cell transfer [analogous to that studied by Barisic et al. (15)] combined with strategies that bypass MHC-I loss and the NK-exclusion microenvironment, rather than checkpoint blockade alone.

There are some limitations to our study. First, the cohort size (33 unique patients, 16 events) limits statistical power for subgroup analyses. The ICI response analysis is exploratory, with 19 evaluable patients, and the upper bound of the CORE odds ratio confidence interval (CI) approaches 1.0. Replication in an independent AM cohort, ideally a subset of AM samples from the TCGA-SKCM Telescope dataset (45), is a priority for subsequent work. Second, HLA class I typing was derived from RNA-seq using OptiType for 25 of 33 patients; typing of the remaining 8 patients using arcasHLA is in progress, and the 100% cohort-level HML6 binder coverage is bounded by the current 25-patient typed subset. Third, antigenicity is assessed only *in silico*. Functional antigenicity requires demonstration of peptide presentation on patient tumors by HLA immunoprecipitation with mass spectrometry and T cell reactivity by tumor-infiltrating lymphocyte assay or MHC multimer staining, neither of which is performed in this study. Fourth, our per-patient mutation burden covariate is derived from the curated somatic coding SNV table presented in the study by Liang et al. (3), which is a rank-preserving proxy rather than a raw call set; full TMB calibration awaits dbGaP-controlled access to the original VCFs. Fifth, the *LIN28A/HMGA2* co-elevation finding is correlational, and a direct demonstration of LIN28A binding to CORE-locus transcripts in AM tissue, for example, by CLIP-seq, would strengthen the mechanistic claim.

In conclusion, our study shows that locus-specific HERV expression can identify a clinically aggressive, mechanistically coherent, and therapeutically relevant subtype of AM. The CORE signature, its *LIN28A/let-7/HMGA2* oncofetal axis association, its inverse relationship to immune checkpoint inhibitor response, and the independent identification of *HML6_20p11.21* as a structurally intact pan-HLA-presentable HERV-K antigen candidate together reframe AM as a disease stratifiable by locus-specific endogenous retroviral expression, and suggest actionable biomarkers and antigen candidates for translational development.

## Data Availability

The original contributions presented in the study are included in the article/Supplementary material, further inquiries can be directed to the corresponding author.

## Supplementary material

SUPPLEMENTARY FIGURE S1 Purity, stromal, and anatomical-site confound diagnostics (relates to R5). **(A)** PC1 of the top-500 variable-feature PCA versus ESTIMATE StromalScore (Spearman ρ with inline stat). PC1 is a stromal-composition axis rather than a biological axis. Multi-sample patients are labeled to show within-patient co-location. **(B)** PC2 versus StromalScore, stratified by anatomical SITE (primary vs metastatic). PC2 captures primary-vs-metastatic biology independently of stromal content. **(C)** -log10(p) of association between each of PC1–PC5 and candidate covariates (SITE, StromalScore, ESTIMATEScore, ImmuneScore, PATIENT_ID, METASTASIZED); display values are color-scaled.

SUPPLEMENTARY FIGURE S2 Feature-class PCA of the AM cohort. (A-D) PCA of the top-500 most variable features restricted to each feature class: **(A)** gene, **(B)** all HERV, **(C)** HERV-K (HML) only, **(D)** LINE-1 (L1). Points colored by SITE, shaped by METASTASIZED status. Different feature classes produce distinct sample embeddings, justifying class-specific downstream analysis. **(E)** PCA of the combined (gene + HERV + L1) feature matrix colored by PATIENT_ID; replicate samples from the same patient (25a/25b, 29a/29c, 34a/34b) co-locate, confirming that within-patient correlation dominates PC1 variance at the sample level.

SUPPLEMENTARY FIGURE S3 Clustering objectivity assessment (relates to R3). **(A)** Kaplan-Meier survival by the original manual three-cluster assignment from the initial submission (PC1/PC2 quadrant labeling). **(B)** KM by data-driven ConsensusClusterPlus k = 2 “Supercluster”. **(C)** KM by data-driven k = 4. **(D)** Boxplot of ESTIMATE StromalScore distribution by consensus cluster (k = 4), showing that cluster identity tracks stromal content. Robust log-rank p-values are reported in-panel; Cox HRs with patient-clustered SE are reported in the main text.
