# Supplementary figures and images for: Locus-specific HERV expression identifies an aggressive, NK-depleted, checkpoint-refractory acral melanoma phenotype
Source: Front Med (Lausanne). 2026 Jul 6;13:1834744. doi: 10.3389/fmed.2026.1834744 (PMC13381639; doi:10.3389/fmed.2026.1834744)

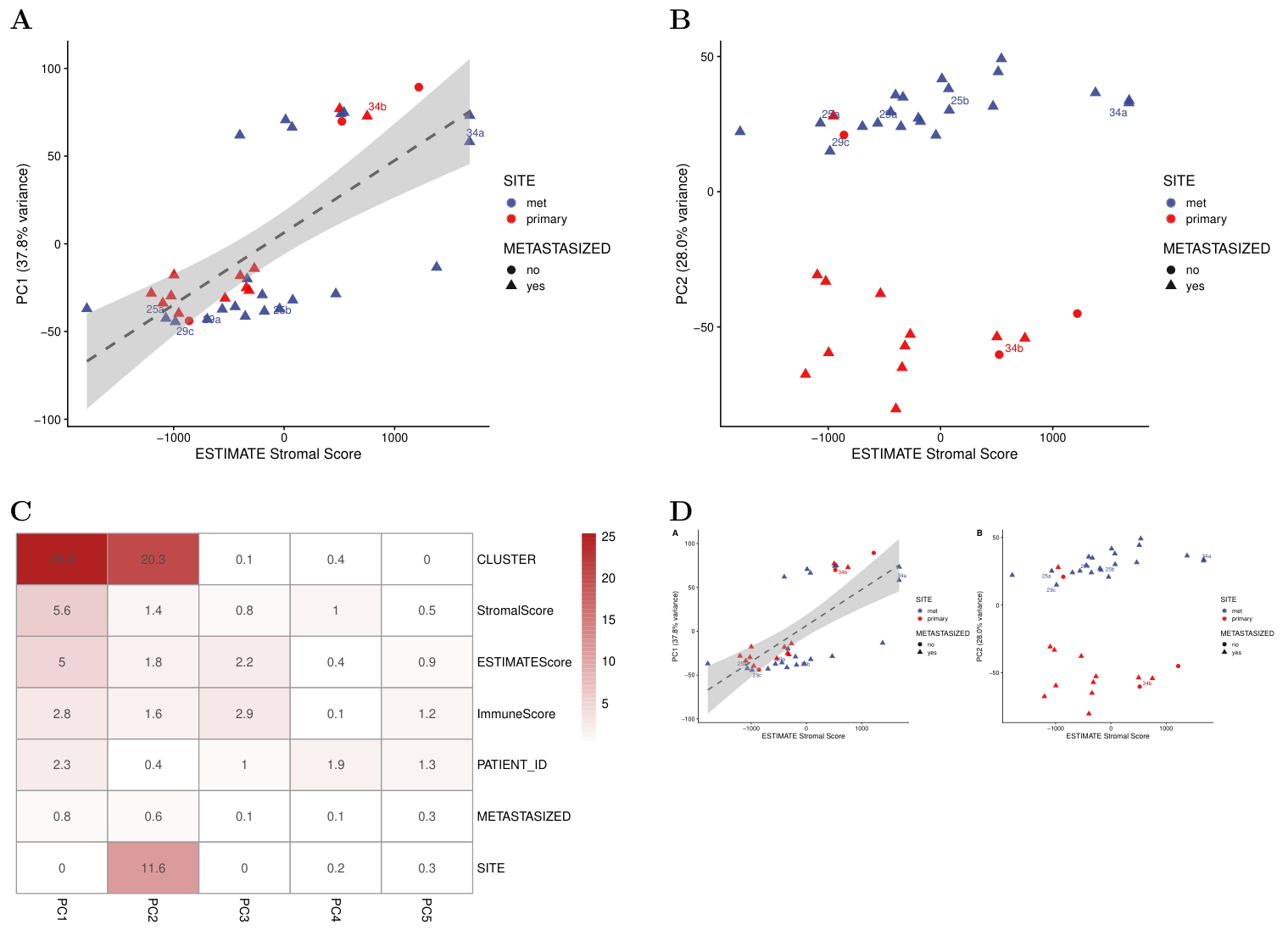

Supplement: SUPPLEMENTARY FIGURE S1 — Purity, stromal, and anatomical-site confound diagnostics (relates to R5). (A) PC1 of the top-500 variable-feature PCA versus ESTIMATE StromalScore (Spearman ρ with inline stat). PC1 is a stromal-composition axis rather than a biological axis. Multi-sample patients are labeled to show within-patient co-location. (B) PC2 versus StromalScore, stratified by anatomical SITE (primary vs metastatic). PC2 captures primary-vs-metastatic biology independently of stromal content. (C) -log10(p) of association between each of PC1–PC5 and candidate covariates (SITE, StromalScore, ESTIMATEScore, ImmuneScore, PATIENT_ID, METASTASIZED); display values are color-scaled. [file Image_1.tiff]

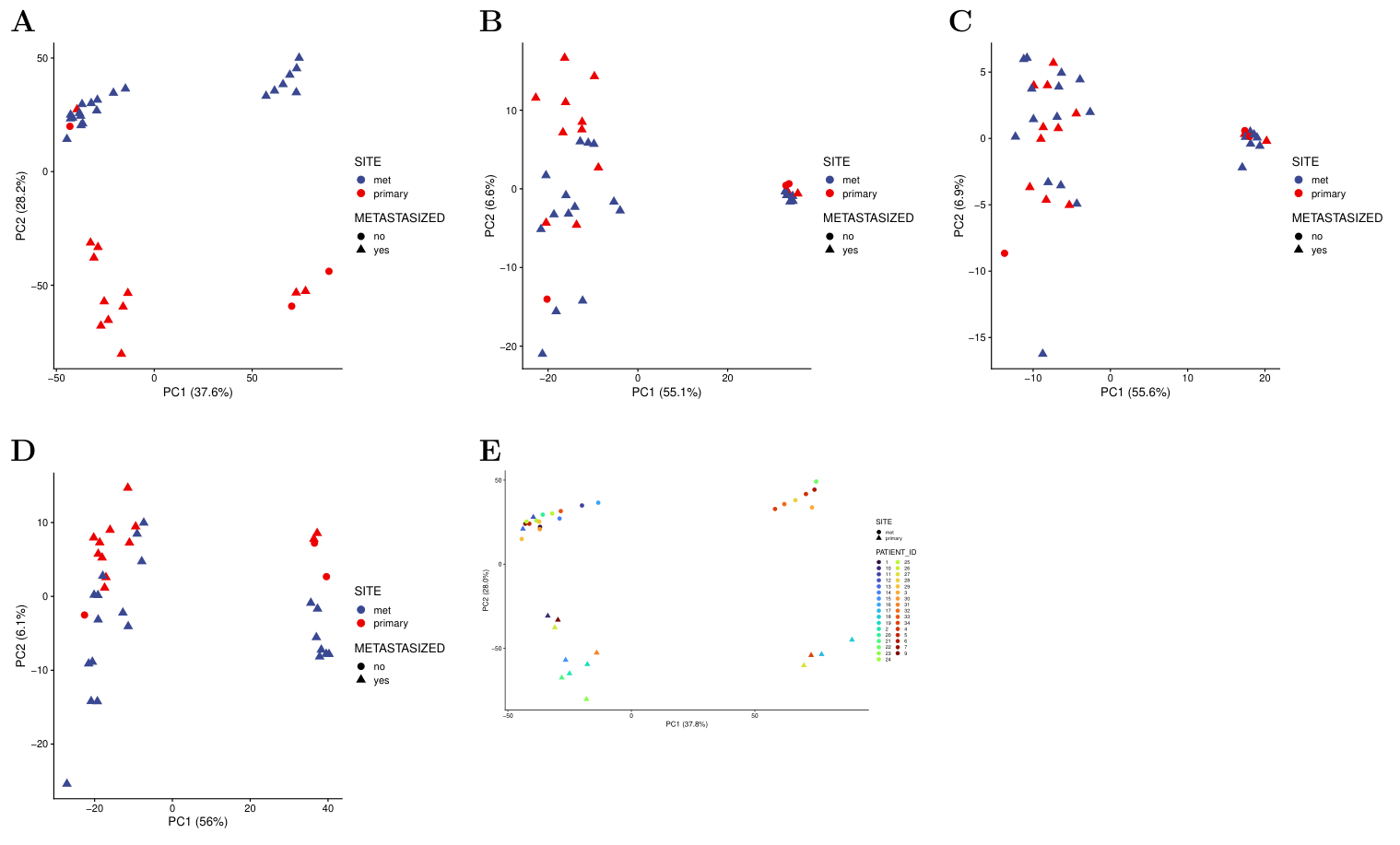

Supplement: SUPPLEMENTARY FIGURE S2 — Feature-class PCA of the AM cohort. (A-D) PCA of the top-500 most variable features restricted to each feature class: (A) gene, (B) all HERV, (C) HERV-K (HML) only, (D) LINE-1 (L1). Points colored by SITE, shaped by METASTASIZED status. Different feature classes produce distinct sample embeddings, justifying class-specific downstream analysis. (E) PCA of the combined (gene + HERV + L1) feature matrix colored by PATIENT_ID; replicate samples from the same patient (25a/25b, 29a/29c, 34a/34b) co-locate, confirming that within-patient correlation dominates PC1 variance at the sample level. [file Image_2.tiff]

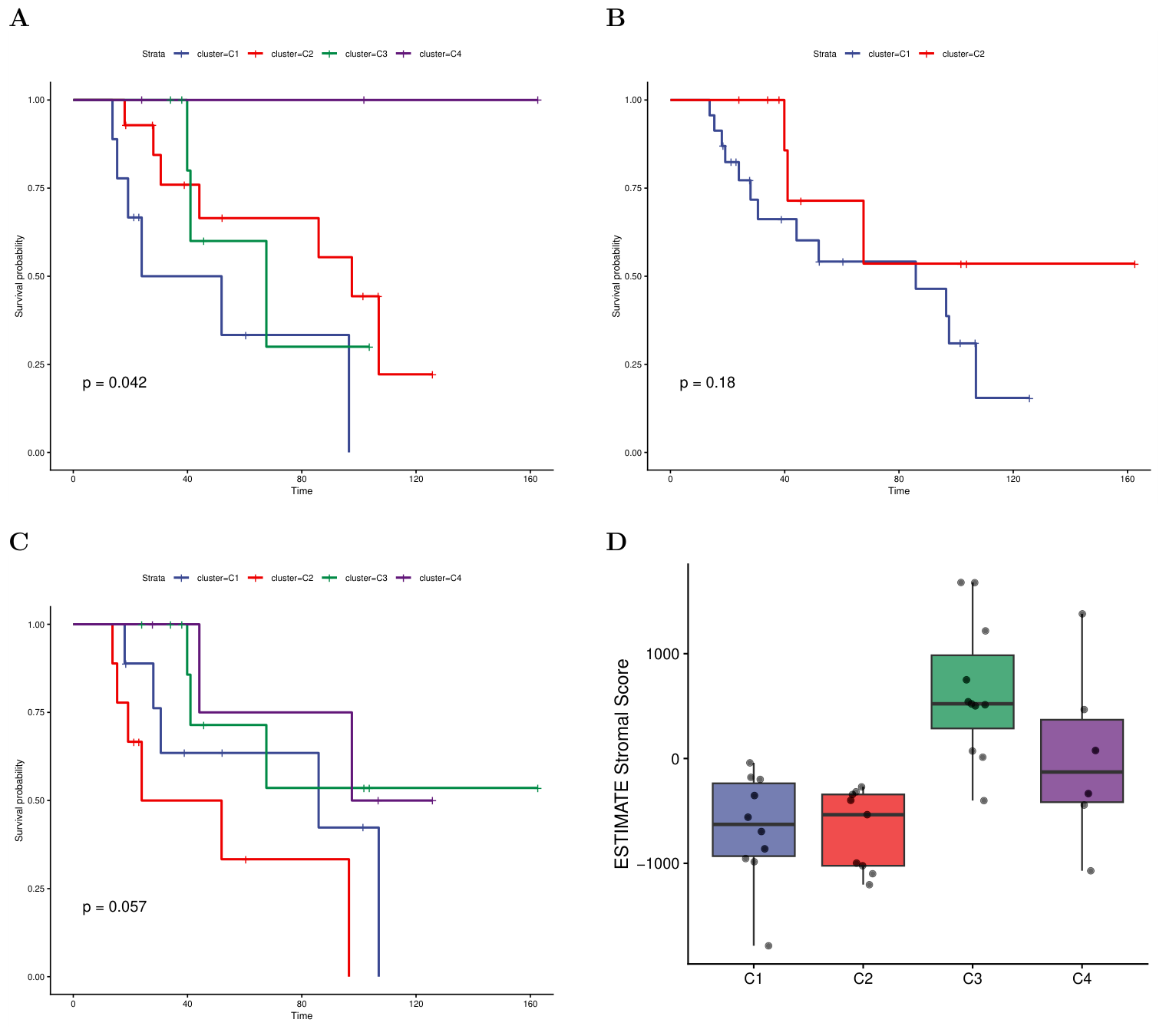

Supplement: SUPPLEMENTARY FIGURE S3 — Clustering objectivity assessment (relates to R3). (A) Kaplan-Meier survival by the original manual three-cluster assignment from the initial submission (PC1/PC2 quadrant labeling). (B) KM by data-driven ConsensusClusterPlus k = 2 “Supercluster”. (C) KM by data-driven k = 4. (D) Boxplot of ESTIMATE StromalScore distribution by consensus cluster (k = 4), showing that cluster identity tracks stromal content. Robust log-rank p-values are reported in-panel; Cox HRs with patient-clustered SE are reported in the main text. [file Image_3.tiff]

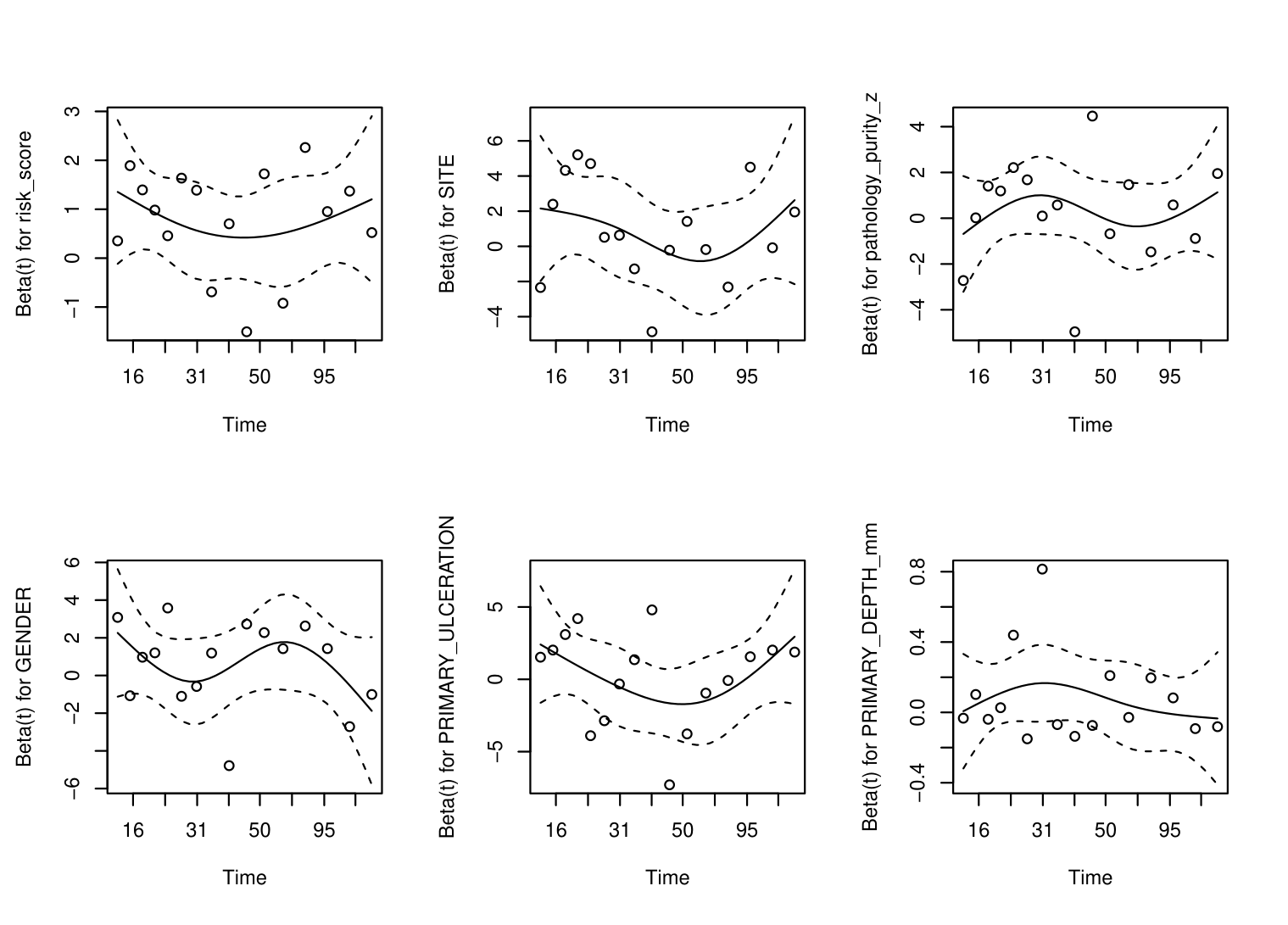

Supplement: Supplementary file 4 [file Image_4.tiff]
